# A comparison of multidimensional qualities discriminant of selection in elite adolescent Australian basketball athletes

**DOI:** 10.1371/journal.pone.0256032

**Published:** 2021-08-13

**Authors:** Jacob Joseph, Fleur McIntyre, Christopher Joyce, Aaron Scanlan, Ashley Cripps

**Affiliations:** 1 School of Health Sciences, University of Notre Dame Australia, Fremantle, West Australia, Australia; 2 Human Exercise and Training Laboratory, School of Health, Medical and Applied Sciences, Central Queensland University, Rockhampton, Queensland, Australia; Instituto Politecnico de Viana do Castelo, PORTUGAL

## Abstract

**Purpose:**

The aims of this study were to (1) quantify the multidimensional attributes of male and female basketball athletes under 16 years of age (U16) and under 18 years of age (U18), and (2) identify attributes that distinguish selection into a talent pathway according to sex and age group.

**Methods:**

67 male and 71 female athletes competing in U16 and U18 selection trials for a state based Australian basketball talent pathway completed a multidimensional testing battery. The test battery consisted of anthropometric, physical (20- linear sprint, countermovement jump height, Yo-Yo Intermittent Recovery Test Level 2), technical (Basketball Jump Shooting Accuracy Test), tactical (video decision making), and psychological (Sports Orientation Questionnaire, Psychological Performance Inventory-Alternative) assessments. Mean differences and independent t-tests were used to assess comparative differences between selected and non-selected athletes within each age and sex cohort. Stepwise discriminant analyses were used to identify attributes that were the strongest discriminators of selection in each group (male U16, male U18, female U16, and female U18).

**Results:**

The discrimminant models showed for male U16 athletes smaller height (ES = -0.18) and greater shooting accuracy (ES = 0.52) was most discriminant of selection. Results were largely homogenous for male U18 athletes with lower visualisation score (ES = -0.62) most discriminant of selection. In female cohorts, faster 20-m sprint time (ES = -0.66) and taller height (ES = 0.58) was most discriminant of selection in U16 athletes while greater shooting accuracy (ES = 0.67), countermovement jump height (ES = 1.04), and height (ES = 0.65) was most discriminant of selection in U18 athletes.

**Conclusions:**

These results emphasise the differing selection priorities within adolescent basketball cohorts according to sex and age group. The testing of anthropometric, physical and technical attributes may hold particular utility in adolescent female basketball given their identified importance to selection across U16 and U18 cohorts.

## Introduction

Talent identification (TID) and talent development practices are used to identify athletes with the greatest performance potential and optimally prepare them for future sporting success [1, 2]. Sporting pathways are typified by a hierarchical progression from inclusive competitions encouraging many participants to increasingly exclusive competitions reserved for athletes with perceived advanced ability or potential for success in senior elite completion [1]. This hierarchical structure enables effective allocation of resources such as coaching, support staff, and training facilities to athletes deemed most likely to excel in the relevant sport [3]. Theoretically, the overarching aim of a talent pathway is to progress key performance capabilities in athletes to a level necessary for professional adult competition [4]. To optimise the talent pathway, there is a clear need to first select athletes with the greatest potential to excel after receiving the development stimulus (e.g. training camps). However, longitudinal examinations of talent pathways in various sports have highlighted poor progression of adolescent athletes into adult competition [5–7], raising concerns regarding the efficacy of talent identification and/or talent development processes used in sports. When examining specific sports, basketball in Australia adopts a hierarchical structure indicative of typical talent pathways [1, 3, 6, 8]. However the selection of athletes at the elite adolescent pathway relies solely on the observation from coaches and selectors during game based scenarios at selection camps, with no traditional TID processes or testing utilised.

It is unclear if or how selection is influenced by the sex and age of athletes in a SPP. In this regard, basketball research has identified differences in match demands between sex [9, 10] and age groups [11]. In turn, these variations in match demands between cohorts are likely underpinned by differences in anthropometric and physical attributes between sexes and age groups. Indeed, anthropometric (height, body mass, and body fat percentage) and physical fitness attributes (jumping ability, linear speed, change-of-direction speed, upper- and lower-body strength, and aerobic capacity) have been shown to improve across age groups consisting of under 18 years of age (U18), under 20 years of age (U20), and senior international-level, male basketball athletes [12]. Furthermore, superior anthropometric (height) and physical fitness attributes (jumping ability, linear speed, change-of-direction speed, and aerobic capacity) have been identified in under 16 years of age (U16) elite male basketball athletes [13] compared to under 14 years of age (U14) elite male basketball athletes [14]. However examination of only anthropometric and physical attributes may not provide a complete understanding of attributes selectors and coaches prioritise in identifying and selecting talented athletes of varying sex and age.

Research has demonstrated that optimal TID models applied in team sports consider a combination of anthropometric, physical, technical, tactical, and psychological attributes [15, 16]. For example, a multidimensional TID applied in Australian football was observed to be 6% more sensitive for player selection than uni-dimensional TID models [16]. Specifically, better technical and physical performance in the form of handballing and dynamic jumping, as well as tactical decision-making ability were the strongest predictors for selection into a U18 male talent pathway in Australian football [16]. Furthermore, research in soccer demonstrated the technical skills of lobbing and juggling were the strongest predictors of selection in elite athletes under 13 years of age (U13) and U14 [15], while, dribbling skills and tactical aspects such as positioning and decision-making most accurately predicted selection in athletes aged 16–18 years [17]. Variations in selection priorities between age groups in youth athletes, may be indicative of a phenomenon termed the ‘snapshot effect’ in which selection is linked to capacities deemed critical to acute competitive performance but may not be considerate of capacities important for long-term pathway success [18]. However it is currently unclear whether this ‘snapshot effect’ is present within adolescent basketball.

To date, no research had examined if and how multidimensional attributes differ between sexes and age groups in adolescent basketball athletes. Therefore, the aim of the current study was to (1) quantify the multidimensional attributes of U16 and U18 male and female athletes in an Australian basketball talent pathway and (2) identify attributes that discriminate selection (i.e. talent) according sex and age group within the talent pathway. In line with other studies examining multidimensional testing of player attributes in adolescent team sport athletes [15, 18–23], it was hypothesised that attributes discriminant of talent would be specific to each cohort (male U16, male U18, female U16, and female U18) in adolescent basketball athletes.

## Methods

### Participants

A total of 158 athletes competing in selection trials for the male and female Western Australian State Performance Program (SPP) U16 and U18 teams were recruited for this study. Only athletes (n = 138) free of injury, available at date of testing and consenting to be part of the study participated in testing protocols. All athletes were part of extended SPP squads that had been previously selected from larger camps. Eight cohort groups were then formed based on selection into the final teams competing at the Australian national championships including selected and non-selected male U16s, male U18s, female U16s, and female U18s. Ethical approval for all procedures was granted by the University of Notre Dame Australia Human Research Ethics Committee (reference # 018062F), with all athletes and their parents/guardians providing written informed consent prior to participating in the study.

### Experimental protocol

Data collection occurred during the fourth week of the 10 week pre-season training phase with all included athletes free from any injury that would have affected their ability to perform the administered test protocols. All anthropometric variables were measured first, followed by a standardised 15-min warm-up involving dynamic stretching, moderate-intensity jogging, and mobility exercises. Each athlete was then randomly assigned to one of five stations including two physical performance stations, as well as technical, tactical, and psychological performance stations following anthropometric assessment. Athletes then rotated through each subsequent station with a 2-min standing rest period between each station. Sport science researchers experienced in delivering the testing protocols were assigned to each station and delivered the same test for the duration of the assessment period to ensure testing consistency. The final physical variable, the Yo-Yo Intermittent Recovery Test Level 2 (YYIR2), was completed one week after the main data collection due to time constraint.

#### Anthropometric testing

Anthropometric attributes included standing height to the nearest 0.1 cm using a stadiometer (Hart Sport, Queensland, Australia) and body mass to the nearest 0.01 kg using electronic scales (Model UC-231, A&D Mercury Pty. Ltd., Tokyo, Japan). In addition, decimal age to the day of testing was determined for each athlete.

#### Physical fitness testing

Physical attributes were assessed using a test battery consisting of 20-m linear sprints, countermovement vertical jumps, and the YYIR2. A 2-min passive standing rest was allocated between each test. Three trials for the 20-m linear sprint and countermovement vertical jump tests were performed and the best measurement was used for analysis. Standardised verbal encouragement was provided for all assessments (e.g. “explode from the floor”, “keep running through the last gate”).

20-m linear sprint time was measured to assess linear speed using electronic timing gates (Swift Performance, Lismore, Australia) positioned at 5 m, 10 m, and 20 m. The lights and reflector for each timing gate were set 1.5 m apart. A 0.5-s correction was added to the raw split times to account for the first propulsive motion occurring prior to initiate timing at the first gate [24]. Athletes completed each sprint starting in an upright position and were instructed to avoid any backward rocking motions upon commencing each sprint. If athletes rocked backwards prior to each sprint, the trial was deemed invalid and the sprint re-attempted. Athletes completed three sprint trials with 2 min of passive standing recovery between each trial. Sprint time was recorded to the nearest 0.01 s and the fastest time for each split, irrespective of which trial they occurred, was used for analysis.

Countermovement vertical jump testing was used to assess lower-body power [25]. Athletes were instructed to perform a countermovement jump and displace the highest vane possible on a Vertec (Swift Performance, Lismore Australia) with their dominant hand. The initial baseline vane reading was determined by each athlete displacing the highest vane possible while reaching overhead standing adjacent to the Vertec. Each athlete performed three jump trials with at least 1 min of passive standing recovery period between trials. Jump height to the nearest 1 cm was taken as the outcome variable.

The YYIR2 was used to assess aerobic capacity in each athlete [26]. Across the test, athletes performed two 20-m runs within each shuttle with a 10-s active recovery between each shuttle. The test consists of stages progressing at increased speeds with a pre-defined number of shuttles in each stage. An audio cue was used to signal the beginning, the turn, and the end within each shuttle and athletes were required to touch the marked line (or reach behind it) with their foot simultaneously or prior to the audio cue to successfully complete that run. Athletes were given one warning if they failed to complete a 20-m run within the allotted time. The final stage and shuttle completed successfully was converted to total distance (m) and used for analysis. The YYIR2 had reduced participation due to less athletes able to attend on this day of testing. The anthropometric and physical fitness testing batteries were chosen due to their wide application in talent selection procedures in adolescent team sports [13, 14, 18] and the strong reliability of each test in adolescent team sport athletes (20-m linear sprint time: coefficient of variation (CV) = 1.5%, intraclass correlation coefficient (ICC) = 0.89 [27]; countermovement vertical jump height: CV = 2.4%, ICC = 0.99 [28]; YYIRT2: CV = 11.0%, ICC = 0.95 [29]).

#### Technical skill testing

The Basketball Jump Shooting Accuracy Test (BJSAT) was utilised to assess shooting accuracy in each athlete. This test has been shown to be reliable (CV = 16.2%, ICC = 0.71) and valid (comparison between two and three-point shots p <0.01) in assessing shooting accuracy in male semi-professional basketball athletes [30, 31]. Athletes were required to shoot the ball from eight different locations including four two-point shots and four three-point shots during the BJSAT. Athletes were instructed to complete the BJSAT as quickly as possible to replicate match-specific time constraints. Each shot was scored as 0 (missed shot that does not touch rim or backboard), 1 (missed shot that contacts the rim or backboard), 2 (made shot which contacts the rim or backboard), or 3 (made shot without touching the rim or backboard). Each athlete completed two trials and the total summed score across all shots in both trials was used for analysis [31]. Prior to completing the BJSAT, athletes were familiarised with test procedures and operation through extensive instruction, demonstration, and a 2-min shooting warm-up. A 2-min passive standing recovery was administered between trials.

#### Tactical ability testing

A video decision-making task was implemented to assess the tactical ability of athletes [16]. Athletes analysed 21 offensive scenarios during basketball game-play to decide the best passing option out of four available options in each scenario. Video footage was taken from publicly available male and female Australian junior national championship games filmed using a camera elevated on the sideline at mid-court. Each video scenario included a lead time of ~5 s prior to the decision-making moment to ensure adequate contextual understanding was acquired by athletes before making a decision. At the decision-making moment, the scenario was paused for 5 s with athletes required to choose the best passing option of those presented. The correct response was determined through the independent responses of three expert coaches (senior Australian state coaches with a minimum of 8 years’ coaching experience) who viewed a total of 50 scenarios. Only video scenarios where agreement was evident between all expert coaches were included in the test. Athletes were tested in an individualised manner using an iPad 2 (Apple Inc., California, United States). All athletes were tested in isolation from their peers. During testing, the video was not able to be paused or replayed for athletes. After three familiarisation video scenarios, each athlete viewed 21 scenarios with a single tactical decision made for each scenario. The total correct responses were tabulated and used for analysis. Due to the limited availability of athletes for testing, only U18 male and female groups completed this assessment.

#### Psychological testing

A psychological assessment consisting of the Sports Orientation Questionnaire (SOQ) [32] and the Psychological Performance Inventory—Alternative (PPI-A) [33] was administered to each athlete. The SOQ contains 32 items with three psychological subsections of ‘competitiveness’, ‘goal’, and ‘win’. The PPI-A contains 14 items which form four psychological subsections of ‘determination’, ‘self-belief’, ‘positive cognition’ and ‘visualisation’. Each question is answered on a Likert scale from 1 (strongly disagree) to 5 (strongly agree), with the total score for each subsection summed across the included questions and used for analysis. The questionnaire was completed individually. The psychological questionnaires were chosen due to their suitable application in youth athletes [34] and within basketball [35] and strong retest reliability in adolescents (SOQ: ‘competitiveness’ [r = 0.89], ‘goal’ [r = 0.73], ‘win’ [r = 0.82] [32]) and athletes (PPI-A: ‘determination’ [r = 0.93], ‘self-belief’ [r = 0.84], ‘positive cognition’ [r = 0.83], and ‘visualisation’ [r = 0.95] [33]).

### Statistical analysis

Mean ± standard deviation were calculated for each variable in each cohort group. Assumption of normality was confirmed using the Kolmogorov-Smirnov test for each variable. Independent t-tests and Hedge’s *g* effect sizes with 95% confidence intervals (CI) were used to assess differences in variables between selected and non-selected groups within each sex and age group (i.e. selected vs. non-selected male U16s, selected vs. non-selected male U18s, selected vs. non-selected female U16s, and selected vs. non-selected female U18s). Hedge’s *g* was interpreted as: trivial, <0.20; small, 0.20–0.59; moderate, 0.60–1.19; large, 1.20–1.99; and very large, ≥2.00 [36].

Stepwise discriminant analyses using a forward entry method was used to determine the strongest variables discriminant of selection in each cohort (i.e. male U16, male U18, female U16, and female U18). Each anthropometric, physical, technical, and tactical variable was included independently, and only athletes undertaking all testing within these dimensions were included in the model. However, YYIR2 data were excluded from the models due to the reduced participation in this test (male U16, n = 37; male U18, n = 27; female U16, n = 42; female U18, n = 26). Due to the larger number of psychological subsets, a separate analysis was conducted only using psychological subsets as independent variables. Each model was assessed for predicted accuracy and variance. Statistical significance was set at p <0.05 with SPSS (version 25.0; IBM SPSS Statistics, Chicago, IL) used for all analyses.

## Results

**Fig 1:**
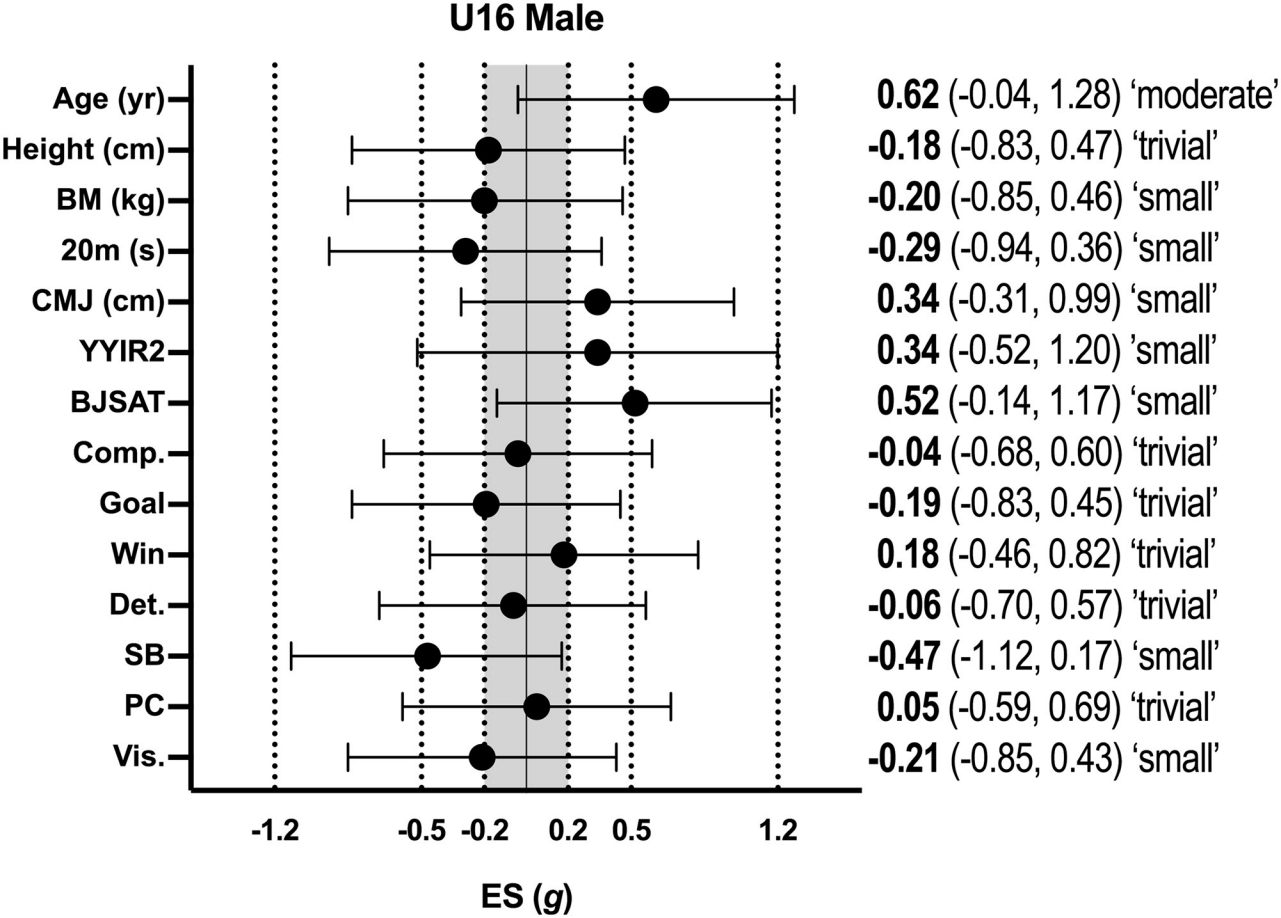
Effect sizes between selected and non-selected male U16 basketballers. BM–body mass; CMJ–countermovement jump; YYIR2 –Yo-Yo Intermittent Recovery Test Level 2; BJSAT–Basketball Jump Shooting Accuracy Test: DM–decision-making; Comp–competitiveness; Det.–determination; SB–self-belief; PC–positive cognition; Vis.–visualisation.

**Fig 2:**
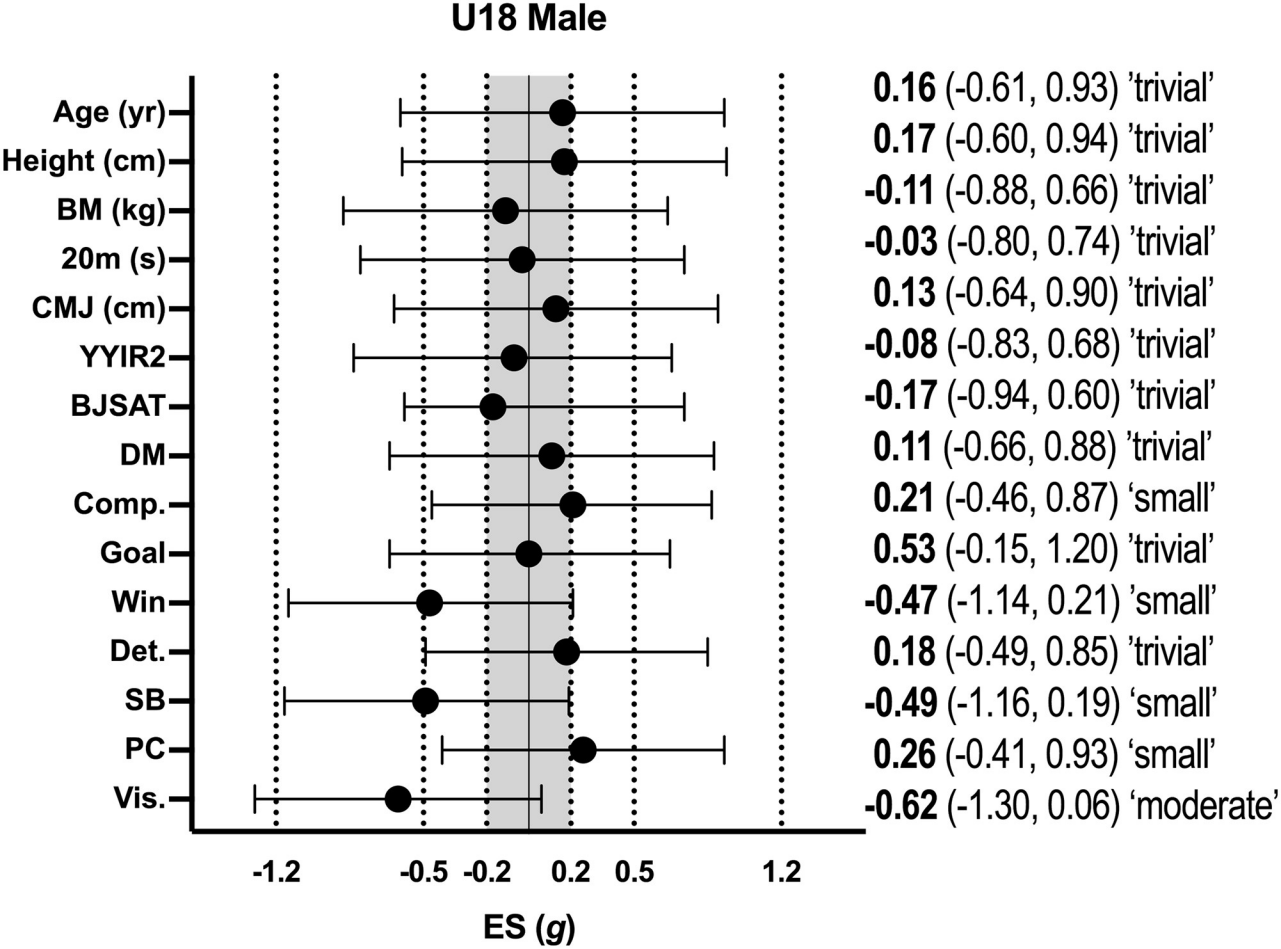
Effect sizes between selected and non-selected male U18 basketballers. BM–body mass; CMJ–countermovement jump; YYIR2 –Yo-Yo Intermittent Recovery Test Level 2; BJSAT–Basketball Jump Shooting Accuracy Test: DM–decision-making; Comp–competitiveness; Det.–determination; SB–self-belief; PC–positive cognition; Vis.–visualisation.

Descriptive statistics (mean ± SD) for all variables according to age group for male basketball athletes are shown in Table 1, with the effect sizes for differences between selected and non-selected athletes in each age group shown in Fig 1 (male U16 athletes) and Fig 2 (male U18 athletes). In male U16 athletes, non-significant differences were evident between selected and non-selected athletes for all variables, with a moderate effect only being reached for age with selected athletes being older than non-selected athletes (p = 0.07; *g* = -0.62; 95% CI = -0.04, 1.28). In male U18 athletes, non-significant differences were evident between selected and non-selected athletes for all variables, with a moderate effect only being reached for the psychological item visualisation with selected athletes possessing lower scores than non-selected athletes (p = 0.06; *g* = -0.62; 95% CI = -1.30, 0.06).

**Table 1:** Mean ± standard deviation anthropometric, physical, technical, tactical, and psychological attributes with statistical comparisons between selected and non-selected male adolescent basketball athletes according to age group.

Variables	Male U16 athletes			Male U18 athletes		
	Selected (n = 18)	Non-selected (n = 19)	P-value	Selected (n = 16)	Non-selected (n = 14)	P-value
***Anthropometric attributes***						
Age (yr)	14.89 ± 0.42	14.63 ± 0.44	0.07	16.21 ± 0.62	16.12 ± 0.42	0.69
Height (cm)	183.1 ± 8.2	184.4 ± 6.2	0.58	188.3 ± 5.9	187.3 ± 6.2	0.67
Body mass (kg)	72.5 ± 12.6	74.8 ± 9.5	0.54	76.8 ± 9.8	77.8 ± 8.3	0.78
***Physical attributes***						
20-m sprint time (s)	3.49 ± 0.16	3.53 ± 0.15	0.37	3.40 ± 0.12	3.41 ± 0.14	0.94
Countermovement jump height (cm)	56.1 ± 10.6	52.8 ± 7.9	0.30	57.2 ± 10.7	55.9 ± 8.0	0.74
YYIR2 distance (m)	443 ± 85	410 ± 109	0.43	465 ± 139	477 ± 190	0.85
***Technical attribute***						
BJSAT score (AU)	24.9 ± 4.3	22.8 ± 3.3	0.11	24.9 ± 3.8	25.6 ± 4.4	0.66
***Tactical attribute***						
Decision-making (correct responses)	NC	NC	NC	13.1 ± 2.0	12.9 ± 2.0	0.78
***Psychological attributes***						
Competitiveness	59.1 ± 3.5	59.3 ± 3.5	0.90	60.6 ± 4.3	59.7 ± 4.7	0.54
Goal	24.4 ± 3.1	25.2 ± 4.0	0.55	26.0 ± 3.7	25.8 ± 4.2	0.99
Win	22.0 ± 5.0	21.2 ± 4.4	0.58	23.4 ± 4.1	25.4 ± 2.6	0.16
Determination	12.3 ± 1.6	12.4 ± 2.1	0.85	13.2 ± 1.7	12.8 ± 2.1	0.60
Self-belief	12.8 ± 1.4	13.9 ± 2.5	0.14	13.3 ± 1.8	14.4 ± 2.6	0.14
Positive cognition	15.9 ± 1.5	15.8 ± 2.1	0.88	16.4 ± 1.7	15.9 ± 2.4	0.44
Visualisation	11.3 ± 2.2	11.8 ± 1.7	0.52	11.4 ± 1.8	12.6 ± 1.8	0.06

*Abbreviations*: U16 –under 16 years of age; U18 –under 18 years of age; YYIR2 –Yo-Yo Intermittent Recovery Test Level 2; BJSAT–Basketball Jump Shooting Accuracy Test; NC–not collected.

**Fig 3:**
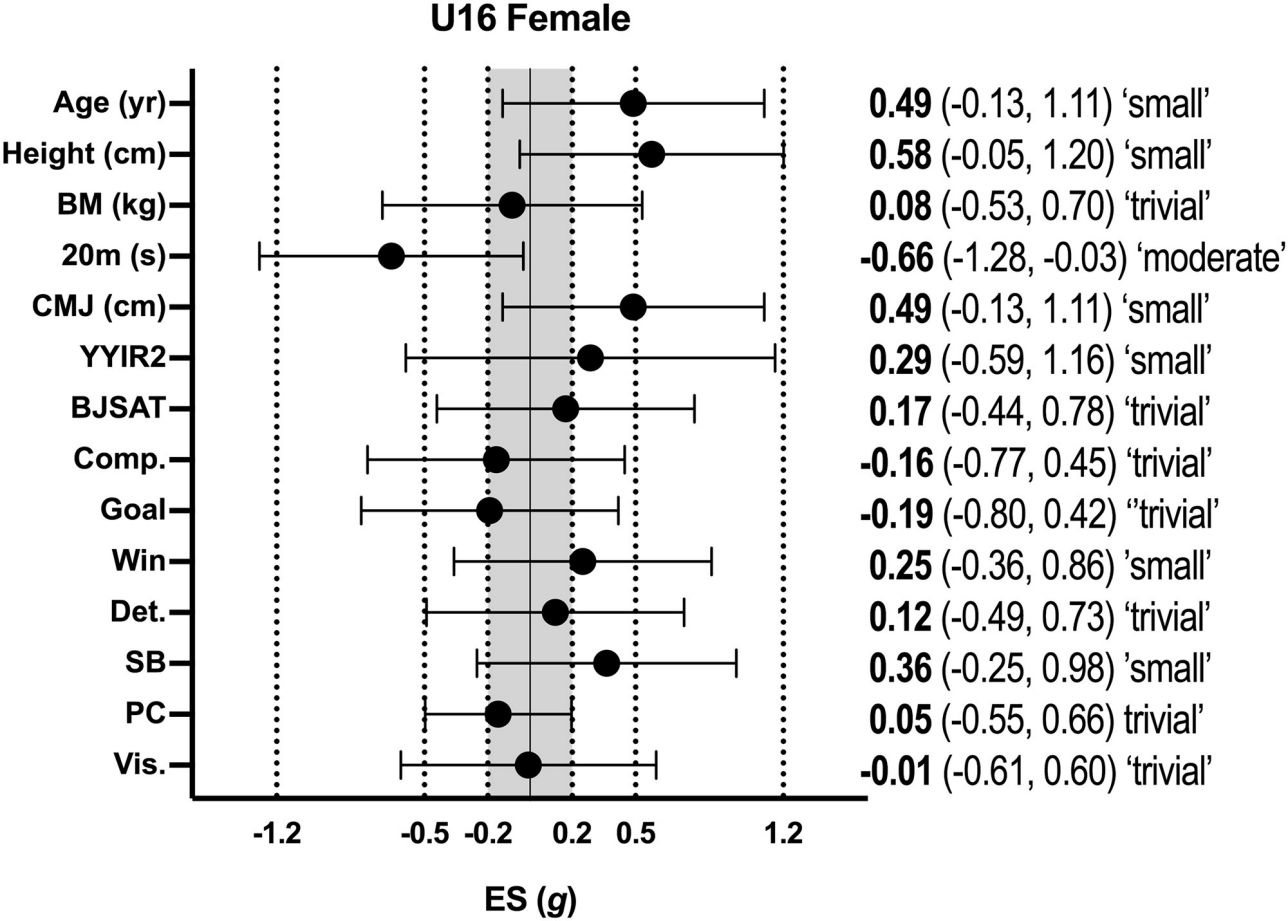
Effect sizes between selected and non-selected female U16 basketballers. BM–body mass; CMJ–countermovement jump; YYIR2 –Yo-Yo Intermittent Recovery Test Level 2; BJSAT–Basketball Jump Shooting Accuracy Test: DM–decision-making; Comp–competitiveness; Det.–determination; SB–self-belief; PC–positive cognition; Vis.–visualisation.

**Fig 4:**
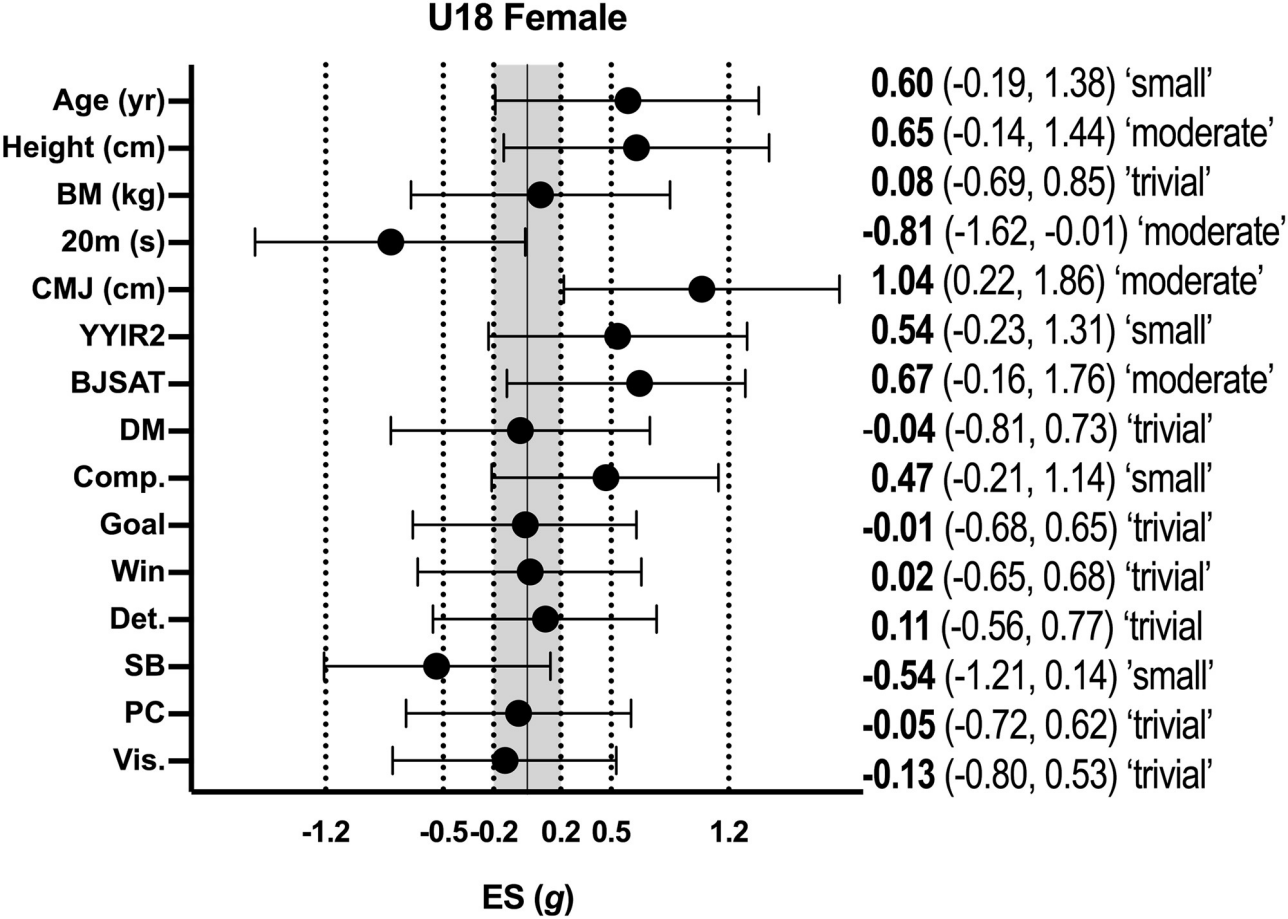
Effect sizes between selected and non-selected female U18 basketballers. BM–body mass; CMJ–countermovement jump; YYIR2 –Yo-Yo Intermittent Recovery Test Level 2; BJSAT–Basketball Jump Shooting Accuracy Test: DM–decision-making; Comp–competitiveness; Det.–determination; SB–self-belief; PC–positive cognition; Vis.–visualisation.

Descriptive statistics (mean ± SD) for all variables according to age group for female basketball athletes are shown in Table 2, with the effect sizes for differences between selected and non-selected athletes in each age group shown in Fig 3 (female U16 athletes) and Fig 4 (female U18 athletes). In female U16 athletes, significant moderate differences for 20-m sprint time were evident with selected athletes being faster than non-selected athletes (p = 0.03; *g* = -0.66; 95% CI = -1.28, -0.03). In female U18 athletes, significant moderate differences were apparent for 20-m sprint time (p = 0.04; *g* = -0.82; 95% CI = -1.62, -0.01), countermovement jump height (p = 0.02; *g* = 1.04; 95% CI = 0.22, 1.86), and shooting accuracy (p = 0.01; *g* = 0.95; 95% CI = 0.14, 1.76), with selected athletes performing better than non-selected athletes.

**Table 2:** Mean ± standard deviation anthropometric, physical, technical, tactical, and psychological attributes with statistical comparisons between selected and non-selected adolescent female basketball athletes according to age group.

Variables	Female U16 athletes			Female U18 athletes		
	Selected (n = 18)	Non-selected (n = 24)	P-value	Selected (n = 13)	Non-selected (n = 16)	P-value
***Anthropometric attributes***						
Age (yr)	14.68 ± 0.54	14.40 ± 0.56	0.11	16.20 ± 0.55	15.89 ± 0.51	0.15
Height (cm)	174.4 ± 6.3	170.4 ± 6.8	0.06	178.9 ± 5.3	175.5 ± 5.1	0.11
Body mass (kg)	64.0 ± 7.6	64.7 ± 8.7	0.82	69.3 ± 5.6	68.5 ± 9.8	0.80
***Physical attributes***						
20-m sprint time (s)	3.74 ± 0.18	3.85 ± 0.13	0.03	3.69 ± 0.18	3.86 ± 0.20	0.04
Countermovement jump height (cm)	43.8 ± 5.9	40.9 ± 5.4	0.11	48.9 ± 6.1	43.3 ± 5.2	0.02
YYIR2 distance (m)	286 ± 75	265 ± 60	0.51	340 ± 106	283 ± 95	0.16
***Technical attribute***						
BJSAT score (AU)	23.1 ± 4.8	22.3 ± 4.7	0.59	24.5 ± 2.9	22.0 ± 3.7	0.01
***Tactical attribute***						
Decision-making (correct responses)	NC	NC	NC	11.5 ± 2.0	11.5 ± 2.0	0.93
***Psychological attributes***						
Competiveness	58.8 ± 6.0	59.6 ± 4.9	0.61	57.6 ± 4.1	54.9 ± 7.1	0.16
Goal	25.6 ± 3.5	26.2 ± 2.9	0.54	23.8 ± 3.0	24.1 ± 4.4	0.82
Win	20.1 ± 5.0	18.9 ± 4.8	0.42	19.0 ± 4.6	18.9 ± 3.8	0.99
Determination	12.9 ± 1.8	12.7 ± 7.9	0.67	12.3 ± 1.9	12.1 ± 1.8	0.76
Self-belief	14.2 ± 2.2	13.4 ± 1.9	0.23	12.6 ± 1.6	13.6 ± 2.1	0.11
Positive cognition	16.4 ± 2.9	16.3 ± 1.9	0.87	15.0 ± 2.2	15.1 ± 2.8	0.88
Visualisation	11.9 ± 2.2	12.0 ± 1.97	0.98	10.7 ± 2.4	11.0 ± 2.3	0.70

*Abbreviations*: U16 –under 16 years of age; U18 –under 18 years of age; YYIR2 –Yo-Yo Intermittent Recovery Test Level 2; BJSAT–Basketball Jump Shooting Accuracy Test; NC–not collected.

Variables retained in the discriminant models of selection are shown in Table 3. In male U16 athletes, height (p = 0.07) and BJSAT score (p = 0.03) were retained, correctly classifying 63.2% of selected athletes. In male U18 athletes, visualisation (p = 0.06) was retained in the model, correctly classifying 65.7% of selected athletes. In female U16 athletes, 20-m sprint time (p = 0.03) and height (p = 0.02) were retained in the model, correctly classifying 66.7% of selected athletes. In female U18 athletes, BJAST score (p = 0.01), countermovement jump height (p = 0.006), and height (p = 0.004) were retained in the model, classifying 73.1% of selected athletes.

**Table 3:** Stepwise discriminant analyses to identify the strongest variables predicting selection into a talent pathway in adolescent basketball athletes according to sex and age group.

Cohort group	Predictor variable(s)	Wilks’ Lambda statistic	Exact F statistic	P-value	Correct classification (%)
**Male**					
***U16***					
1	Height	0.911	3.42	0.07	63.2
2	BJSAT score	0.817	3.80	0.03	
***U18***					
1	Visualisation	0.898	3.76	0.06	65.7
**Female**					
***U16***					
1	20-m sprint time	0.888	5.05	0.03	66.7
2	Height	0.815	4.43	0.02	
***U18***					
1	BJSAT score	0.758	7.66	0.01	73.1
2	CMJ height	0.643	6.38	0.01	
3	Height	0.555	5.89	0.00	

*Abbreviations*: U16 –under 16 years of age; U18 –under 18 years of age; BJSAT–Basketball Jump Shooting Accuracy Test; CMJ–countermovement jump.

## Discussion

In basketball and other youth team sports, previous studies have predominantly examined anthropometric and physical attributes explanatory of selection into talent pathways [19, 22–24, 38, 39]. Fewer studies have incorporated technical [15, 17, 19] and psychological [20, 37] dimensions, and fewer still have included a tactical dimension [17]. Therefore it was the aim of this study, to utilise a multidimensional testing battery when identifying selection priorities in adolescent basketball athletes according to sex and age group. The findings of this study support the hypothesis that the variables discriminating between selected and non-selected athletes as well as explaining selection into a talent pathway would be specific to basketball athlete cohorts based on sex and age. In turn, our findings may add further support for the snapshot approach in TID research [15, 18].

When exploring our results according to cohort group, some contradictory findings were evident in the male U16 athletes examined in our study compared to existing findings. Specifically, smaller stature (ES = -0.18) was discriminant of selection in the U16 male cohort. Contrary to this finding, height is significantly positively associated with offensive rating in playoff games [40] and team ranking for point guards, shooting guards, and small forwards [38] in professional male basketball athletes given it can be advantageous in executing several fundamental match activities such as rebounding, shooting, and shot blocking [38]. Furthermore, elite male U14 basketball athletes have been shown to be significantly taller than their sub-elite (p = 0.004) [14] and non-elite counterparts (p <0.005) [39]. Similarly, elite male U16 basketball athletes have been demonstrated to be significantly taller than non-elite male U16 basketball athletes (p <0.001) [14], with increased height being identified as a significant predictor of athlete ranking in elite male U16 basketball athletes (p <0.001) [13]. It could be hypothesised that the smaller stature in selected male U16 athletes we observed could highlight differing selection and tactical priorities of the coaches in the SPP we investigated, potentially employing the popularised ‘small ball’ tactical style of play. ‘Small ball’ is characterised by teams consisting of shorter than average players to increase floor spacing and subsequently three-point attempts to maximise scoring [40]. Interestingly, jump shooting accuracy (ES: 0.52) was the only other variable (in addition to height) retained in the discriminant model of selection in male U16 athletes. Together, these analyses suggest the tactical direction of ‘small ball’ was likely driving selection priorities in the male U16 cohort specifically.

Unlike the U16 cohort, largely homogenous results were evident in the male U18 athletes we examined. Only the psychological subset of visualization (lower scores) (ES = -0.62) was retained in the discriminant model of selection in male U18 athletes. This finding is contradictory to past research [41] which proposes the psychological attribute of visualisation or imagery aids in skill development and ability to perform in elite sporting competition (i.e. judo, curling, and javelin). Psychological assessments have rarely been incorporated into basketball TID research; however, findings reported in starting male collegiate basketball athletes demonstrated a stronger relationship (R^2^ = 0.41) between mental toughness and basketball performance (measured using PERF statistic) than starting female collegiate basketball athletes (R^2^ = 0.34) [35]. Interestingly, psychological variables did not differentiate selection status in any other cohort apart from male U18 athletes in our study. This in conjunction with the dearth of research exploring the role of psychological attributes in pathway selection within basketball make the practical applications of this finding unclear. As such, future research should further explore the importance of psychological outcomes and their role in pathway selection.

In opposition to the male cohorts, pronounced significant differences were evident between selected and non-selected in the female cohorts. Specifically, height (ES = 0.58) and 20-m linear sprint time (ES = -0.66) discriminated between selected and non-selected female U16 athletes. These findings are consistent with the limited research in female U16 basketball athletes showing height and physical fitness attributes of vertical jump height (p <0.001), 20-m sprint time (p = 0.013), and multi-stage fitness test level (p = 0.05) as significant predictors of match performance as rated by independent coaches [13]. Furthermore, higher ranked teams had significantly greater height (p = 0.029) and physical performance represented by repeated sprint dribble performance time (discriminant ratio coefficient [DRC] = 0.44), medicine ball throw (DRC = 0.38), and 20-m sprint time (DRC = 0.20) than lower ranked teams in elite female U15 basketball athletes [42]. In further support of our findings regarding the importance of lower-body power and speed in adolescent female basketball athletes, linear sprint time and repeated sprint time have been shown to be positively associated with the number of assists (linear sprint time, p <0.01; repeated sprint time, p <0.01) and steals (linear sprint time, p <0.05; repeated sprint time, p <0.01) achieved during match-play in elite female U16 and U18 basketball athletes [43].

Height was the only consistent attribute that discriminated between selected and non-selected athletes in both U16 (ES = 0.58) and U18 (ES = 0.65) female cohorts. However, in contrast to U16 athletes, countermovement jump height (ES = 1.04) and shooting accuracy (ES = 0.67) were superior in selected female U18 athletes compared to non-selected female U18 athletes. The combination of superior height and countermovement jump height could allude to selection priorities favoring abilities which aid in the execution of key match tasks such as rebounding and contesting shots from opponents [44]. Additionally, greater height and countermovement jump height along with superior jump shooting accuracy highlight the importance of creating vertical space to minimise the influence of shot blocking attempts from opponents and create greater opportunity for scoring. Furthermore, the magnitude of differences in variables between selected and non-selected athletes were greatest in the female U18 cohort than any cohort examined in our study. The pronounced differences observed in female U18 athletes may be attributed to variations in basketball participation rates and the subsequent size of the talent pool between sexes. With the most recent available statistics evidencing in 2017 vastly greater participation rates in basketball of Australian male youth (8.6%) compared to female youth (5.9%) [45], and 2012 numbers of Australian male youth (212,700) compared to female youth (83,200) [46]. Nevertheless, the results of the current study outline physical and technical attributes may be more important to develop and identify for selection into talent pathways among adolescent female basketball athletes than their male counterparts.

It is notable that the findings of this study, that factors discriminant of selection change between sex-matched levels of a talent pathway, is in line with previous literature [3, 18, 19, 22]. Such changes may allude to a phenomenon previously termed a ‘snapshot approach’ to talent identification [18]. This practice preferentially identifies those who possess attributes acutely favored by coaches or that are deemed important for immediate competition success, but not necessarily favorable for athlete retention in subsequent stages of the talent pathway. This talent identification approach may account for the poor retention of athletes frequently reported in many sports talent pathways [5–7], with relatively few athletes progressing from initial stages of talent pathways through to subsequent stages. For example, in the Australian Football talent pathway it was demonstrated that only 25% of athletes progressed through each stage of the talent pathway from initial pathway selection at 15 years of age through to adult professional recruitment [6]. Further longitudinal research is required to confirm if the factors discriminant of talent change as athletes progress through subsequent stages of the talent pathway or if they remain static.

The limitations of this study should be recognised when interpreting the presented findings. Firstly, given only one SPP was investigated (Western Australia) the results may not be representative of SPP in other states and territories in Australia. Accordingly, future research should look to incorporate other SPP’s across Australia when conducting similar research to provide holistic analyses of selection processes and priorities according to sex and age group in adolescent basketball athletes. Secondly, due to the small sample size able to be recruited in the selected SPP, large variability was evident in many attributes which may have limited the accuracy and applicability of the effect sizes and discrimminant models generated. Thirdly, even though we utilised a multidimensional testing battery, other approaches to assessment potentially useful in basketball settings were not incorporated into our study. For example, there has been an increased call for in-situ tasks and stronger ecological validity within assessments selected in TID processes [47]. Consequently, development of testing protocols assessing performance during small-sided games or in-situ tasks where match skills can be directly quantified or rated may be useful to explore in future basketball research on this topic. Finally, this study employed a cross-sectional design which prohibits assessment of longitudinal development and success of selected athletes. Future research should aim to assess the longitudinal selection priorities and development of athletes in basketball TID programs to further understand sex and age differences among adolescent athletes. The findings of our study also highlight the need to identify attributes that coaches perceive as important for selection, which has not been incorporated in traditional TID protocols.

## Conclusion

In male athletes, data demonstrated smaller height and superior shooting accuracy discriminated selection in U16 athletes, while lower visualisation was important for selection in U18 athletes. In female athletes, data demonstrated taller height and faster 20-m linear sprint time discriminated selection in U16 athletes, while taller height, better shooting accuracy, and higher countermovement jump height were important for selection in U18 athletes. Our study is the first to use a multidimensional approach in identifying attributes important for selection into a talent pathway in adolescent basketball athletes according to sex and age group. The results of this study provide further evidence of a ‘snapshot approach’ occurring within talent identification processes. Such talent identification decisions may lead to poor athlete retention at subsequent stages of the pathway and long-term athlete development outcomes. Further longitudinal research is required to establish if factors discriminant of adolescent talent change as athletes progress through subsequent stages of the talent pathway or if they remain static.

## Supporting information

S1 Dataset (XLSX)
